# Prediction mode of more than 5 central lymph nodes metastases in clinically node-negative ipsilateral papillary thyroid carcinoma with tumor size 1 to 4 cm

**DOI:** 10.1097/MD.0000000000019809

**Published:** 2020-04-17

**Authors:** Lei Jin, Hai-Li Sun, Liang Zhou, Lei Xie, Yi-Yu Zhuang, Jian-Biao Wang

**Affiliations:** a Department of Head and Neck Surgery; b Nursing Department, the Affiliated Sir Run Run Shaw Hospital, Zhejiang University School of Medicine #3 East Qingchun Road, Hangzhou, Zhejiang, PR China.

**Keywords:** central lymph node metastasis, papillary thyroid carcinoma, prediction model, tumor size

## Abstract

According to the 2015 American Thyroid Association guidelines, either lobectomy or total thyroidectomy was recommended for patients with papillary thyroid carcinoma (PTC) of 1 to 4 cm without extrathyroidal extension and lymph node metastasis. However, lymph node metastases showed strong association with recurrence and low survival rate, especially in PTC patients with more than 5 metastatic lymph nodes. Therefore, this study aimed to investigate the predictive factors of more than 5 central lymph nodes metastases (CLNM) in PTC patients with tumor sizes of 1 to 4 cm. A total of 382 patients with clinically node-negative (cN0) ipsilateral PTC who underwent thyroidectomy with central neck dissection between January 2012 and December 2016 were retrospectively analyzed. CLNMs of >5 were found in 54 (14.1%) patients, while CLNM was detected in 230 (60.2%) patients. Multivariate logistic regression revealed age < 45 years (*P* < .001), male gender (*P* = .013), and tumor sizes of >2 cm (*P* = .001) as independent predictive factors of >5 CLNMs in cN0 ipsilateral PTC patients with tumor sizes 1 to 4 cm. The prediction equation (Y = 1.694 × age + 0.807 × gender + 1.190 × tumor size − 3.530) was developed, with a sensitivity (57.4%) and a specificity (80.8%), respectively, at an optimal cut-off point of −1.685. Therefore, if the predictive value was higher than −1.685 according to the equation in cN0 ipsilateral PTC patients with tumor sizes 1 to 4 cm, then total thyroidectomy might be considered.

## 1 Introduction

Due to the indolent course and excellent prognosis of papillary thyroid carcinoma (PTC), there is no definite agreement regarding the initial treatment for PTC, which ranged from active observation alone to total thyroidectomy with radioactive iodine (RAI) therapy.^[1–3]^ According to the 2009 American Thyroid Association (ATA) guidelines,^[4]^ lobectomy is only recommended for PTCs <1 cm without any extrathyroidal extension (ETE) and clinical evidence of lymph node metastasis. Total thyroidectomy has been recommended as the initial surgical procedure for PTCs >1 cm. However, according to the revised 2015 ATA guidelines^[5]^ either lobectomy or total thyroidectomy is recommended for PTCs between 1 and 4 cm without ETE and clinical evidence of lymph node metastasis.

Cervical lymph node metastasis is relatively common in patients with PTC.^[6,7]^ Even in patients with clinically node-negative (cN0) PTCs, central lymph node metastasis is found in 60.9%.^[8]^ Lymph node metastasis showed association with local recurrence and low survival rate,^[9–11]^ especially in PTC patients with 5 or more metastatic lymph nodes.^[12]^ Sugitani et al have reported that the risk of recurrence is significantly higher in patients with 5 or more lymph nodes metastases (19%) when compared to those with <5 metastases (8%).^[12]^ According to the ATA modified 2009 recurrence risk stratification system, PTC patients with more than 5 metastatic lymph nodes are considered as high-risk group.^[5]^ Also the latest National Comprehensive Cancer Network (NCCN) guidelines on thyroid cancer recommended completion thyroidectomy for ipsilateral PTC patients who had lobectomy plus isthmusectomy when the patient had more than 5 metastatic lymph nodes.^[13]^ Furthermore, RAI therapy is typically recommended for PTC patients with >5 positive lymph nodes according to the latest NCCN guidelines on thyroid cancer.^[13]^ Therefore, there is a need for a model to estimate the risk prediction of more than 5 central lymph nodes metastases (CLNM) in cN0 ipsilateral PTC patients with tumor sizes 1 to 4 cm, so that they are optimally removed, and minimize the rate of completion thyroidectomy.

Hence, in this study, the incidence and risk factors for more than 5 CLNMs in a group of cN0 ipsilateral PTC patients with tumor sizes of 1 to 4 cm were investigated. A prediction model was then developed to estimate the risk of more than 5 CLNMs and facilitate therapeutic decision for ipsilateral PTC resection.

## 2 Materials and methods

### 2.1 Patients

In total, 382 cN0 PTC patients whose tumor size ranged from 1 cm to 4 cm without ETE and underwent total thyroidectomy or lobectomy with central neck dissection (CND) between January 2012 and December 2016 were included in this retrospective study. The medical records of these patients were retrospectively reviewed based on a prospectively collected database from the Department of Head and Neck Surgery in the Affiliated Sir Run Run Shaw Hospital, Zhejiang University School of Medicine. The exclusion criteria were as follows: patients with non-PTCs (follicular/medullary/anaplastic), tumor size <1 cm or >4 cm, ETE, bilateral carcinoma, tumors located in the isthmus, clinically apparent metastatic disease to nodes (cN1), distant metastasis, or with a history of previous thyroid surgery. A cN1 neck was defined as patients suspected with lymph node metastasis in central or lateral neck by ultrasonography and computed tomography or proved by ultrasound-guided fine needle aspiration (FNA) preoperatively.

Thyroid nodules, as well as central and lateral neck lymph nodes were evaluated using ultrasound examination in each patient. FNA was then used to confirm the malignancy or rule out metastasis of suspicious primary lesions in the lateral cervical lymph nodes. Vocal cord function was assessed by direct or indirect laryngoscopy. In addition, the levels of thyroid hormone, thyroid peroxidase antibody (TPOAb), thyroglobulin antibody (TgAb), parathyroid hormone, calcitonin, and serum calcium were also measured in each patient.

This study was approved by the Ethical Committee of the Affiliated Sir Run Run Shaw Hospital, Zhejiang University School of Medicine.

### 2.2 Surgical methods

According to the 2009 ATA guidelines,^[4]^ total thyroidectomy was performed in all patients included in this study from 2012 to 2015, and was mainly applied for thyroid cancers >4 cm, cN1, or distant metastasis based on the 2015 ATA guidelines,^[5]^ and most of the patients included in this study after that received lobectomy in 2016. Ipsilateral prophylactic central neck dissection (pCND) was routinely recommended for patients. CND is mainly regarded as a level VI dissection according to the ATA guidelines, and unilateral CND involves the removal of prelaryngeal, pretracheal, and single paratracheal nodal basins.^[14]^

All the procedures included were conventional open surgeries and were performed by the same group of senior surgeons. Thyroidectomy was performed by using the technique of capsular dissection as suggested by Thompson et al.^[15]^ Recurrent laryngeal nerves and all parathyroid glands were routinely identified and preserved under direct vision. The vascular supply of the parathyroid glands was confirmed by fine needle pricking test. Devascularized parathyroid gland was excised into tiny fragments and was autotransplanted into the contralateral sternocleidomastoid muscle.

### 2.3 Clinicopathological factors

The clinicopathological factors included in this study were sex, age, coexistent thyroid disease (chronic lymphocytic thyroiditis, CLT), the state of TPOAb and TgAb, maximal tumor size, tumor location based on ultrasound, and multifocal/solitary lesions. The maximal tumor size, multifocal/solitary lesions and tumor location were all determined through ultrasound examination preoperatively. The location of primary thyroid tumor (the largest tumor for multifocal lesions) was categorized into upper, middle, and lower third of the thyroid lobes as described previously.^[8]^ Pathological examination was conducted to diagnose CLT. The normal ranges for serum TPOAb and TgAb were 0 to 5.61 IU/mL and 0 to 4.11 IU/mL, respectively, at our institution. Thyroid autoimmune antibody status was considered positive if the serum level was over the upper range. The status of thyroid autoimmune antibody was classified into four groups:

(1) TPOAb single positive (TPOAb+);
(2) TgAb single positive (TgAb+);
(3) TPOAb and TgAb double positive (TAb+); and
(4) TPOAb and TgAb double negative (TAb−).

### 2.4 Statistical analysis

Statistical analyses were performed using SPSS version 20.0 (IBM, Armonk, NY). Continuous variables are presented as means (standard deviation, SD) or medians (range). Categorical variables are presented as number of cases with percentages (%). Continuous variables were compared using Student's *t* test or Mann–Whitney *U* test, while categorical variables were compared with Pearson chi-square test. The coefficient, odds ratio (OR), and 95% confidence intervals (CI) for relationships between each variable and CLNM (>5/≤5) were calculated using binary logistic regression. *P* < .05 was considered to be statistically significant.

## 3 Results

### 3.1 Patient characteristics

The clinicopathological characteristics of 382 ipsilateal PTC patients with tumor sizes of 1 to 4 cm are shown in Table 1. Of all the 382 PTC patients, 287 (75.1%) were women and 95 (24.7%) were men, with a mean age of 41.6 years (range, 14–73 years), and 225 (58.9%) were <45 years. All patients were diagnosed with primary PTC by intraoperative frozen section examination. In addition, 91 patients had CLT. Three different surgical procedures were performed in all patients:

(1) lobectomy (including isthmectomy and pyramidal lobectomy) with ipsilateral CND (right 49 patients and left 65 patients);
(2) total thyroidectomy with ipsilateral CND (right 127 patients and left 99 patients); and
(3) total thyroidectomy with bilateral CND (42 patients).

**Table 1:**
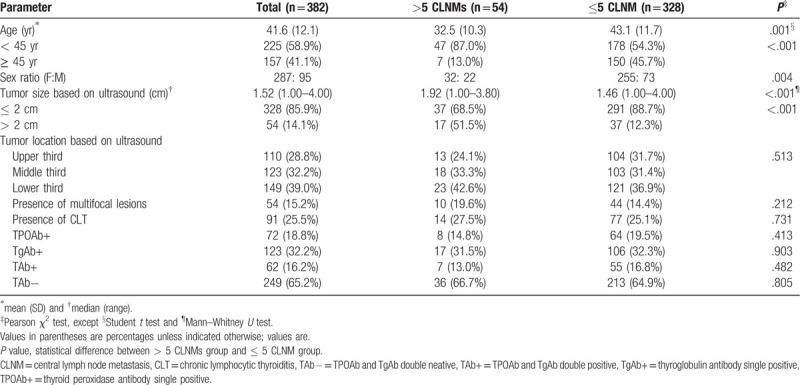
Patient characteristics.

The median number of harvested central lymph nodes was 11.0 (range 0–43), and the median number of metastatic central lymph nodes was 1.0 (range 0–16). CLNMs of >5 were found in 54 (14.1%) patients, while CLNM was detected in 230 (60.2%) patients. None of the patients had any history of head and neck radiation before surgery, and none of them had distant metastasis.

### 3.2 Predictive factors for >5 CLNMs in cN0 ipsilateral PTC patients with tumor sizes of 1 to 4 cm

The associations between clinicopathological characteristics and >5 CLNMs in 382 cN0 PTC patients with tumor sizes 1 to 4 cm were analyzed by univariate and multivariate logistic regression (Table 2). In univariate analysis, age <45 years (*P* < .001), male gender (*P* = .004), and primary tumor size of >2 cm (*P* < .001) showed significant association with more than 5 CLNMs. Other factors, including tumor location, multifocal tumor, coexisting CLT, and the status of thyroid autoimmune antibody showed no signification correlation with >5 CLNMs (Table 2). Multivariate logistic regression analysis confirmed that age <45 years (OR = 5.439, *P* < .001), male gender (OR = 2.241, *P* = .013), and primary tumor size of > 2 cm (OR = 3.288, *P* = .001) as independent predictive factors for >5 CLNMs in cN0 ipsilateral PTC patients with tumor sizes of 1 to 4 cm (Table 3). The predictive efficiency of each independent risk factor was listed in Table 4.

**Table 2:**
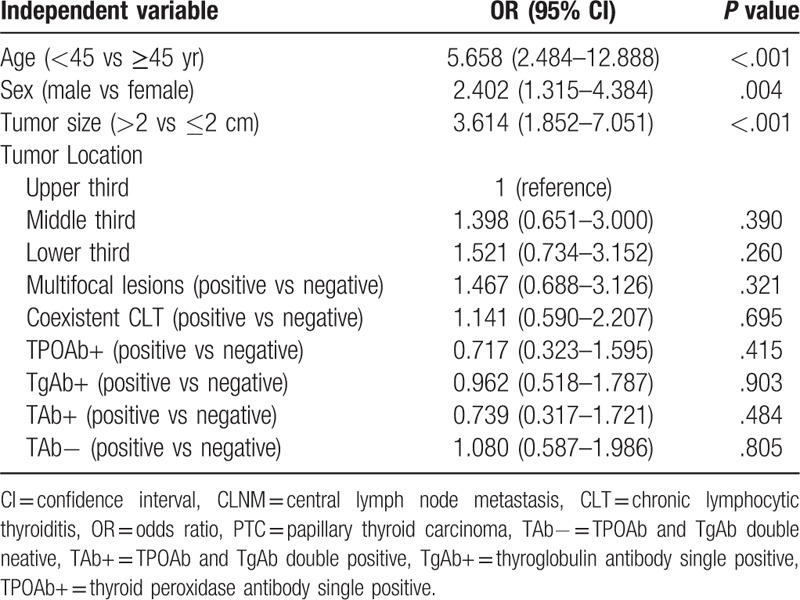
Univariate logistic regression analyses of >5 CLNMs in cN0 ipsilateral PTC patients with tumor sizes of 1 to 4 cm.

**Table 3:**
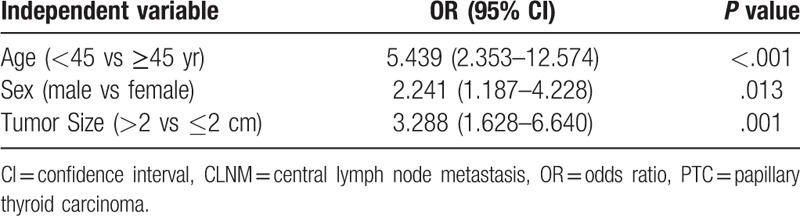
Multivariate logistic regression analyses of >5 CLNMs in cN0 ipsilateral PTC patients with tumor sizes of 1 to 4 cm.

**Table 4:**
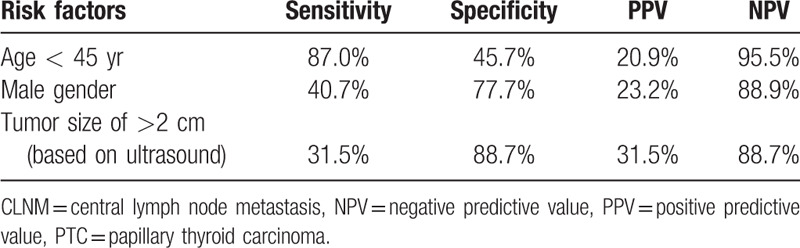
Predictive efficiency of each independent risk factor for >5 CLNMs in cN0 ipsilateral PTC patients with tumor sizes of 1 to 4 cm.

### 3.3 Prediction model of >5 CLNMs in cN0 ipsilateral PTC patients with tumor sizes of 1 to 4 cm

Using the coefficients obtained from multivariate logistic regression analysis, the following prediction equation for more than 5 CLNMs was derived, in which the categorical variables were coded as “1” if present (younger than 45 years, male gender, and tumor size of > 2 cm) and “0” if absent:

Y = 1.694 × age + 0.807 × gender + 1.190 × tumor size − 3.530, where Y means the chance of a positive of > 5 CLNMs.

**Figure 1:**
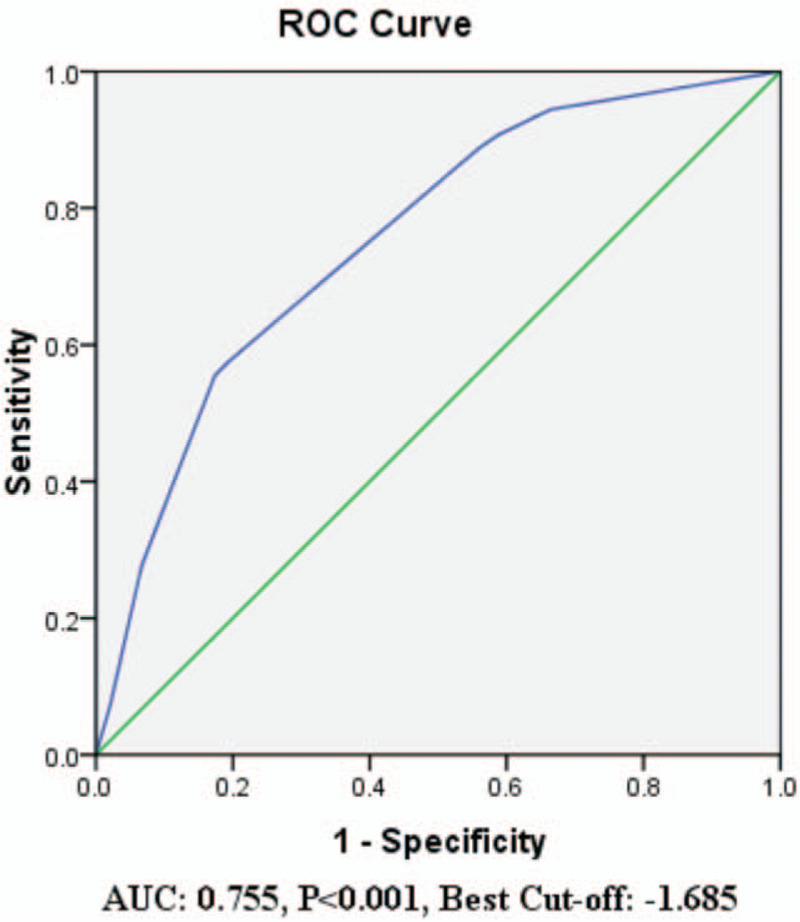
The ROC analysis of cut-off point for Y value in predicting metastases of more than 5 central lymph nodes.

Consequently, the ROC curve analysis was used to determine the optimal cut-off point of Y (range, −3.530 to 0.161) for predicting >5 CLNMs in cN0 ipsilateral PTC patients with tumor sizes of 1 to 4 cm (Fig. 1). The optimal cut-off point was selected as −1.685, which meant that patients with a predictive value of higher than −1.685 had the maximum likelihood to have more than 5 CLNMs. The sensitivity, specificity, positive predictive value (PPV), and negative predictive value (NPV) were 57.4%, 80.8%, 33.0%, and 92.0%, respectively. The Hosmer–Lemeshow test showed no significance (*P* = .901), indicating a good fit of the model.

## 4 Discussion

In contrast to the 2009 ATA guidelines,^[4]^ the 2015 ATA guidelines recommended either lobectomy or total thyroidectomy for ipsilateral PTCs without ETE and clinical evidence of lymph node metastasis.^[5]^ When lobectomy is performed, the usage of RAI therapy is of great concern for PTC patients with high-volumes of (i.e., >5 metastatic lymph nodes) CLNMs. Multivariate adjusted analyses from SEER suggested that RAI treatment improved the overall survival in node-positive adult patients with PTCs.^[16]^ According to the latest NCCN guidelines on thyroid cancer, RAI therapy is typically recommended for PTC patients with >5 positive lymph nodes.^[13]^ The 2015 ATA guidelines also recommended RAI adjuvant therapy after total thyroidectomy in patients with ATA intermediate-risk level PTC.^[5]^ Therefore, the 382 surgically proven cN0 ipsilateral PTC patients with tumor sizes 1 to 4 cm were analyzed to investigate the predictive factors of more than five CLNMs. Better knowledge regarding the predictive factors of >5 CLNMs from this study suggested a more selective approach to identify cN0 ipsilateral PTC patients with the necessity of total thyroidectomy.

In this study, the prevalence of CLNM and more than 5 CLNMs was found to be 60.2% (230 of 382) and 14.1% (54 of 382), respectively. Univariate and multivariate analyses revealed that age <45 years, male gender, and primary tumor size >2 cm as independent predictive factors for more than 5 CLNMs in cN0 ipsilateral PTCs with tumor sizes of 1 to 4 cm.

Age is considered to be an important prognostic factor in patients with PTC. Previous studies have found that age <45 years as a significant risk factor for CLNM in cN0 PTC patients.^[17–18]^ Furthermore, Miyauchi et al have found that <40 years of age as the only significant risk factor for both tumor size enlargement and novel appearance of lymph node metastasis in low-risk papillary thyroid microcarcinomas (PTMCs) during active surveillance.^[19]^ The study also showed that the proportion of appearance of novel lymph node metastasis in PTMC patients those who are younger than 40 years group remained the highest (16.1%) after 10 years observation.^[19]^ In our study, patients with >5 CLNMs were significantly younger than those with ≤5 CLNM (32.5[10.3] vs 43.1 [11.7], *P* < .001). Age <45 years showed significant association with increased risk of > 5 CLNMs in cN0 ipsilateral PTC patients with tumor sizes 1 to 4 cm.

Male gender has been identified as a risk factor for thyroid carcinoma.^[20]^ Several studies have revealed that men exhibited poorer prognosis than women among PTC patients.^[21,22]^ In addition, our previous study showed male gender as an independent predictive factor for non-small-volume central lymph node metastases (more than 5 or ≥2 mm) in cN0 PTC patients.^[23]^ This study also showed male gender as the predictive factor for more than 5 CLNMs in cN0 ipsilateral PTC patients with tumor sizes 1 to 4 cm.

Tumor size is regarded as an important factor in TNM staging for PTC, and larger tumors tend to be more aggressive.^[24]^ Previous studies have shown significant association of tumor size with CLNM, but no consensus was reached on the best cut-off point. Ito^[25]^ and Sun^[17]^ have reported that tumor size of >2 cm is the strongest predictor of CLNM in PTC, while Bozec,^[26]^ Choi,^[27]^ and Koo^[28]^ have reported that tumor size of >1 cm is associated with CLNMs in PTC. In our study, tumor size in patients with >5 CLNMs was significantly greater than in those with ≤5 CLNM [1.92 (1.00–3.80) vs 1.46 (1.00–4.00) cm, *P* < .001]. To predict the risk of more than five CLNMs before operation, the size of the tumor was determined through ultrasound examination preoperatively in this study. Logistic regression analysis showed that tumor size of >2 cm was significantly associated with more than 5 CLNMs in cN0 ipsilateral PTC patients with tumor size of 1 to 4 cm.

The pathological results revealed the existence of multifocal lesions as a risk factor for CLNM in PTC^[17]^ and PTMC.^[18]^ Recently, some studies also showed multifocality as the risk factor of high-volume (i.e., >5 metastatic lymph nodes) CLNMs in PTC.^[23,29]^ However, multifocal lesions according to ultrasonography showed no significant relation with the presence of >5 CLNMs in cN0 ipsilateral PTC patients with tumor sizes 1 to 4 cm in our study. The results of our study were in accordance with those reported by Huang et al, wherein the multifocal lesions determined by preoperative ultrasound were not considered as risk factor for high-volume CLNMs in cN0 PTMC.^[30]^ This might be due to the small malignant nodules (less than 2 mm) that cannot be found during ultrasound examination.

The antigen-specific humoral immune responses induced by TPOAb and TgAb showed association with the development and prognosis of PTC. However, the association between thyroid autoimmune antibody and lymph node metastasis still remained controversial and the existing data are conflicting. Vasileiadis et al have reported that the rate of lymph node metastasis in PTC patients with positive TgAb showed a significant increase when compared with the rate of PTC patients with negative TgAb.^[31]^ Shen et al have demonstrated that PTC patients with positive thyroid autoimmune antibody have more metastatic central compartment lymph nodes.^[32]^ Recently, Wen et al have reported that the antibody status (TPOAb and TgAb double negative, TPOAb and TgAb double positive, and TgAb single positive) acts as an independent risk factor for CLNM in PTC patients with CLT.^[33]^ However, some investigators reported conflicting results to this. Donangelo et al have found that a larger number of cervical lymph nodes were excised in the TgAb positive group than in the TgAb negative group, but the number of metastatic cervical lymph nodes was similar.^[34]^ Shen et al have also found that single-TgAb positivity and co-positivity of TgAb and TPOAb, but not single TPOAb positivity as protective factors of distant metastasis of PTC.^[32]^ Paparodis et al have reported that high TPOAb titers protected against PTC in patients with CLT.^[35]^ In this study, the status of thyroid autoimmune antibody demonstrated no significant relation with > 5 CLNMs in ipsilateral cN0 PTC patients with tumor sizes 1 to 4 cm. The differences in the inclusion criteria of patients, detection methodologies and positive cut-off value might contribute to the differences in the results.

To determine the subset of cN0 ipsilateral PTC patients with tumor sizes 1 to 4 cm who are prone to burden of more than 5 CLNMs before operation, a prediction mode based on independent risk factors was developed. ROC curve analysis was used to determine the cut-off point of predictive value and found that the predictive value of higher than −1.685 as the strongest predictor of more than 5 CLNMs in cN0 ipsilateral PTC patients with tumor sizes 1 to 4 cm. The sensitivity and specificity were 57.4% and 80.8%, respectively, which was similar with, and even greater than the efficacy of ultrasonography in detecting CLNM of PTC.^[36–38]^

However, the present study has several limitations. First, there are inherent features of a non-randomized retrospective cohort study. Second, in our center, ipsilateral pCND was performed routinely for cN0 ipsilateral PTCs, but 42 patients in this study underwent bilateral pCND. Thus, the extent of pCND in this study included unilateral and bilateral clearance, which might influence the number of metastatic lymph nodes in the central compartment, as the prevalence of contralateral paratracheal lymph node metastasis has been reported to range from 3.9% to 30.6% in unilateral cN0 PTC.^[8,28,39–43]^ Third, the patients included in this study, was not very large. Nevertheless, this study has several significant strengths. First, the size of the tumor in this study was determined through preoperative ultrasound examination. Therefore, all the risk factors determined in this study can be detected before operation. Second, an equation has been developed according to the predictive factors, which demonstrated better predictive efficiency than each independent risk factor. Third, only surgically proven data were used, which included only cN0 ipsilateral PTC cases.

In conclusion, this study demonstrated that age <45 years, male gender, and tumor size of >2 cm as strong indicators for more than 5 CLNMs in cN0 ipsilateral PTC patients with tumor sizes 1 to 4 cm. Based on this data, a mathematical model was proposed to quantitatively predict more than 5 CLNMs. Therefore, when the predictive value was higher than −1.685 according to the equation (Y = 1.694 × age + 0.807 × gender + 1.190 × tumor size − 3.530) in cN0 ipsilateal PTC patients with tumor sizes 1 to 4, total thyroidectomy might be considered.

## Author contributions

**Conceptualization:** Lei Jin, Hai-Li Sun, Lei Xie, Yi-Yu Zhuang, Jian-Biao Wang.

**Data curation:** Lei Jin, Hai-Li Sun, Liang Zhou.

**Formal analysis:** Lei Jin, Hai-Li Sun.

**Investigation:** Lei Jin, Yi-Yu Zhuang.

**Validation:** Liang Zhou, Jian-Biao Wang.

**Writing – original draft:** Lei Jin, Hai-Li Sun, Liang Zhou.

**Writing – review & editing:** Lei Jin, Lei Xie, Yi-Yu Zhuang, Jian-Biao Wang.

## References

[1] Wu AW Nguyen C Wang MB What is the best treatment for papillary thyroid microcarcinoma? Laryngoscope 2011;121:1828–9. 21997725 10.1002/lary.22033

[2] Udelsman R Shaha AR Is total thyroidectomy the best possible surgical management for well-differentiated thyroid cancer? Lancet Oncol 2005;6:529–31. 15992702 10.1016/S1470-2045(05)70247-3

[3] Guerrero MA Clark OH Controversies in the management of papillary thyroid cancer revisited. ISRN Oncol 2011;2011:303128. 22091417 10.5402/2011/303128 PMC3197013

[4] American Thyroid Association Guidelines Taskforce on Thyroid Nodules and Differentiated Thyroid Cancer, Cooper DS, Doherty GM, Haugen BR, et al. Revised American Thyroid Association management guidelines for patients with thyroid nodules and differentiated thyroid cancer. Thyroid 2009;19:1167–214. 19860577 10.1089/thy.2009.0110

[5] Haugen BR Alexander EK Bible KC 2015 American Thyroid Association management guidelines for adult patients with thyroid nodules and differentiated thyroid cancer: the American Thyroid association guidelines task force on thyroid nodules and differentiated thyroid cancer. Thyroid 2016;26:1–33. 26462967 10.1089/thy.2015.0020 PMC4739132

[6] Grebe SK Hay ID Thyroid cancer nodal metastases: biologic significance and therapeutic considerations. Surg Oncol Clin N Am 1996;5:43–63. 8789493

[7] Kouvaraki MA Shapiro SE Fornage BD Role of preoperative ultrasonography in the surgical management of patients with thyroid cancer. Surgery 2003;134:946–54. discussion 954-955. 14668727 10.1016/s0039-6060(03)00424-0

[8] Wada N Duh QY Sugino K Lymph node metastasis from 259 papillary thyroid microcarcinomas: frequency, pattern of occurrence and recurrence, and optimal strategy for neck dissection. Ann Surg 2003;237:399–407. 12616125 10.1097/01.SLA.0000055273.58908.19 PMC1514312

[9] Podnos YD Smith D Wagman LD The implication of lymph node metastasis on survival in patients with well-differentiated thyroid cancer. Am Surg 2005;71:731–4. 16468507 10.1177/000313480507100907

[10] Pelizzo MR Boschin IM Toniato A Papillary thyroid carcinoma: 35-year outcome and prognostic factors in 1858 patients. Clin Nucl Med 2007;32:440–4. 17515749 10.1097/RLU.0b013e31805375ca

[11] Zaydfudim V Feurer ID Griffin MR The impact of lymph node involvement on survival in patients with papillary and follicular thyroid carcinoma. Surgery 2008;144:1070–7. 19041020 10.1016/j.surg.2008.08.034

[12] Sugitani I Kasai N Fujimoto Y A novel classification system for patients with PTC: addition of the new variables of large (3 cm or greater) nodal metastases and reclassification during the follow-up period. Surgery 2004;135:139–48. 14739848 10.1016/s0039-6060(03)00384-2

[13] National Comprehensive Cancer Network. NCCN Clinical Practice Guidelines in Oncology (NCCN Guideline^®^). Thyroid Carcinoma. Version 1. 2019. Available at https://www.nccn.org/professionals/physician_gls/pdf/thyroid.pdf. 1 April 2020.

[14] American Thyroid Association Surgery Working Group, American Association of Endocrine Surgeons, American Academy of Otolaryngology-Head and Neck Surgery, American Head and Neck Society, Carty SE, Cooper DS, Doherty GM, et al. Consensus statement on the terminology and classification of central neck dissection for thyroid cancer. Thyroid 2009;19:1153–8. 19860578 10.1089/thy.2009.0159

[15] Thompson NW Olsen WR Hoffman GL The continuing development of the technique of thyroidectomy. Surgery 1973;73:913–27. 4703492

[16] Ruel E Thomas S Dinan M Adjuvant radioactive iodine therapy is associated with improved survival for patients with intermediate-risk papillary thyroid cancer. J Clin Endocrinol Metab 2015;100:1529–36. 25642591 10.1210/jc.2014-4332 PMC4399282

[17] Sun W Lan X Zhang H Risk factors for central lymph node metastasis in cN0 papillary thyroid carcinoma: a systematic review and meta-analysis. PLoS One 2015;10:e0139021. 26431346 10.1371/journal.pone.0139021 PMC4592212

[18] Zhang L Wei WJ Ji QH Risk factors for neck nodal metastasis in papillary thyroid microcarcinoma: a study of 1066 patients. J Clin Endocrinol Metab 2012;97:1250–7. 22319042 10.1210/jc.2011-1546

[19] Miyauchi A Clinical trials of active surveillance of papillary microcarcinoma of the thyroid. World J Surg 2016;40:516–22. 26744340 10.1007/s00268-015-3392-y PMC4746213

[20] Hegedus L Clinical practice. The thyroid nodule. N Engl J Med 2004;351:1764–71. 15496625 10.1056/NEJMcp031436

[21] Shaha AR Shah JP Loree TR Risk group stratification and prognostic factors in papillary carcinoma of thyroid. Ann Surg Oncol 1996;3:534–8. 8915484 10.1007/BF02306085

[22] Besic N Pilko G Petric R Papillary thyroid microcarcinoma: prognostic factors and treatment. J Surg Oncol 2008;97:221–5. 18050283 10.1002/jso.20935

[23] Wang JB Sun YY Shi LH Predictive factors for non-small-volume central lymph node metastases (more than 5 or ≥ 2 mm) in clinically node-negative papillary thyroid carcinoma. Medicine (Baltimore) 2019;98:e14028. 30608456 10.1097/MD.0000000000014028 PMC6344183

[24] Amin MB Edge SB Greene FL 2017 AJCC Cancer Staging Manual. Eighth ed New York: Springer; 2017.

[25] Ito Y Fukushima M Higashiyama T Tumor size is the strongest predictor of microscopic lymph node metastasis and lymph node recurrence of N0 papillary thyroid carcinoma. Endocr J 2013;60:113–7. 22972223 10.1507/endocrj.ej12-0311

[26] Bozec A Dassonville O Chamorey E Clinical impact of cervical lymph node involvement and central neck dissection in patients with papillary thyroid carcinoma: a retrospective analysis of 368 cases. Eur Arch Otorhinolaryngol 2011;268:1205–12. 21607578 10.1007/s00405-011-1639-2

[27] Choi YJ Yun SJ Kook SH Clinical and imaging assessment of cervical lymph node metastasis in papillary thyroid carcinomas. World J Surg 2012;34:1494–9. 10.1007/s00268-010-0541-1 20372903

[28] Koo BS Choi EC Yoon YH Predictive factors for ipsilateral or contralateral central lymph node metastasis in unilateral papillary thyroid carcinoma. Ann Surg 2009;249:840–4. 19387316 10.1097/SLA.0b013e3181a40919

[29] Liu CH Liu YW Zhang L Risk factors for high-volume lymph node metastases in cN0 papillary thyroid microcarcinoma. Gland Surg 2019;8:550–6. 31741886 10.21037/gs.2019.10.04 PMC6842761

[30] Huang XP Ye TT Zhang L Sonographic features of papillary thyroid microcarcinoma predicting high-volume central neck lymph node metastasis. Surg Oncol 2018;27:172–6. 29937168 10.1016/j.suronc.2018.03.004

[31] Vasileiadis I Boutzios G Charitoudis G Thyroglobulin antibodies could be a potential predictive marker for papillary thyroid carcinoma. Ann Surg Oncol 2014;21:2725–32. 24595799 10.1245/s10434-014-3593-x

[32] Shen C Zhang X Qiu Z Thyroid autoimmune antibodies in patients with papillary thyroid carcinoma: a double-edged sword. Endocrine 2017;58:176–83. 28884426 10.1007/s12020-017-1401-7

[33] Wen XZ Wang BW Jin QM Thyroid antibody status is associated with central lymph node metastases in papillary thyroid carcinoma patients with Hashimoto's thyroiditis. Ann Surg Oncol 2019;26:1751–8. 30937662 10.1245/s10434-019-07256-4

[34] Donangelo I Walts AE Bresee C Lymphocytic thyroiditis is associated with increased number of benign cervical nodes and fewer central neck compartment metastatic lymph nodes in patients with differentiated thyroid cancer. Endocr Pract 2016;22:1192–8. 27732096 10.4158/E151078.OR

[35] Paparodis R Imam S Todorova-koteva K Hashimoto's thyroiditis pathology and risk for thyroid cancer. Thyroid 2014;24:1107–14. 24708347 10.1089/thy.2013.0588 PMC4080848

[36] Ito Y Tomoda C Uruno T Clinical significance of metastasis to the central compartment from papillary microcarcinoma of the thyroid. World J Surg 2006;30:91–9. 16369721 10.1007/s00268-005-0113-y

[37] Mulla M Schulte KM Central cervical lymph node metastases in papillary thyroid cancer: a systematic review of imaging-guided and prophylactic removal of the central compartment. Clin Endocrinol (Oxf) 2012;76:131–6. 21722150 10.1111/j.1365-2265.2011.04162.x

[38] Kim SK Woo JW Park I Computed tomography-detected central lymph node metastasis in ultrasonography node-negative papillary thyroid carcinoma: is it really significant? Ann Surg Oncol 2017;24:442–9. 27624581 10.1245/s10434-016-5552-1

[39] Roh JL Kim JM Park CI Central lymph node metastasis of unilateral papillary thyroid carcinoma: patterns and factors predictive of nodal metastasis, morbidity, and recurrence. Ann Surg Oncol 2011;18:2245–50. 21327454 10.1245/s10434-011-1600-z

[40] Eun YG Lee YC Kwon KH Predictive factors of contralateral paratracheal lymph node metastasis in papillary thyroid cancer: prospective multicenter study. Otolaryngol Head Neck Surg 2014;150:210–5. 24367047 10.1177/0194599813514726

[41] Ji YB Yoo HS Song CM Predictive factors and pattern of central lymph node metastasis in unilateral papillary thyroid carcinoma. Auris Nasus Larynx 2016;43:79–83. 26441368 10.1016/j.anl.2015.09.005

[42] Wei T Chen R Zou X Predictive factors of contralateral paratracheal lymph node metastasis in unilateral papillary thyroid carcinoma. Eur J Surg Oncol 2015;41:746–50. 25882035 10.1016/j.ejso.2015.02.013

[43] Lee KE Chung IY Kang E Ipsilateral and contralateral central lymph node metastasis in papillary thyroid cancer: patterns and predictive factors of nodal metastasis. Head Neck 2013;35:672–6. 22715063 10.1002/hed.23016

